# Parental knowledge, attitudes, and practices on probiotic use in preschool children in Serbia: a cross-sectional study

**DOI:** 10.3389/fimmu.2025.1601876

**Published:** 2025-09-01

**Authors:** Maja Đanić, Nebojša Pavlović, Tijana Ostojić, Nikolija Marković, Bojan Stanimirov, Slavica Lazarević, Momir Mikov

**Affiliations:** ^1^ Department of Pharmacology, Toxicology and Clinical Pharmacology, Faculty of Medicine, University of Novi Sad, Novi Sad, Serbia; ^2^ Department of Pharmacy, Faculty of Medicine, University of Novi Sad, Novi Sad, Serbia; ^3^ Department of Fundamental Sciences, Faculty of Technical Sciences, University of Novi Sad, Novi Sad, Serbia; ^4^ Faculty of Medicine, University of Novi Sad, Novi Sad, Serbia; ^5^ Department of Biochemistry, Faculty of Medicine, University of Novi Sad, Novi Sad, Serbia

**Keywords:** probiotics, gut microbiota, children, parents, KAP questionnaire, pharmacomicrobiomics

## Abstract

**Introduction:**

Considering the crucial role of the gut microbiome in children’s immunity and overall health, there is increasing interest in the use of probiotics for children. Insufficient parental awareness may result in the underuse of probiotics in appropriate clinical situations, improper strain selection, incorrect therapy duration, and overlooking potential drug interactions, all of which can undermine their efficacy and safety. Therefore, this study aimed to assess parents’ knowledge, attitudes, and practices regarding probiotic use in preschool-aged children in Serbia, along with the factors influencing these aspects.

**Materials and methods:**

The study was conducted using an anonymous electronic survey distributed via social media to parents of children aged 1–7 years in Serbia. A binary logistic regression model was used to analyze the factors associated with parental knowledge and attitudes toward probiotic use.

**Results:**

The study included 1,625 parents. The median knowledge score of all respondents was 7.0 (interquartile range [IQR]: 6–8), while the median attitude score was 26.0 (IQR: 23–29), based on their respective scales (0–10 for knowledge and 7–35 for attitude) with significant differences (*p* < 0.001) between parents who used probiotics for their preschool children in the past year (62.5%) and those who did not (37.5%). The most common indications for probiotic use were alongside antibiotic therapy reported by 75.2% of parents, and for gastrointestinal issues, stated by 69.3% of parents. Parents showed the least knowledge about the strain-specific effects of probiotics and their interactions with medications. Although overall attitudes were moderately positive, approximately 50% of parents expressed doubts about probiotic efficacy. Logistic regression analysis revealed that higher parental education, occupation related to health care, longer duration of probiotic use, and consideration of probiotic strain selection significantly increased the odds of having adequate knowledge and a positive attitude toward probiotics.

**Conclusion:**

The study revealed that the majority of parents lack adequate knowledge about probiotics and exhibit some skepticism regarding their effectiveness, which is reflected in their practical use for their children. Therefore, the role of healthcare professionals and pediatricians is crucial in educating parents about probiotics, offering guidance on their benefits, proper usage, and the selection of the most appropriate products.

## 1 Introduction

The role of probiotics in children’s immunity and overall health has garnered significant attention in recent years. Probiotics, defined as live microorganisms that confer health benefits to the host when administered in adequate amounts, are known to play a crucial role in maintaining the balance of gut microbiota, which is essential for proper digestive function and immune response. The colonization of the gut microbiota begins at birth and continues to evolve during early childhood, with the first three years being crucial for its maturation (1). Recent studies indicate that the intrauterine environment is non-sterile, suggesting that fetal intestines may be colonized even prenatally. This underscores the importance of a healthy maternal environment for optimal microbiome development from the earliest stages of *in utero* development (2). The composition of the intestinal microbiome, which serves as a distinct fingerprint unique to each individual, is influenced by a variety of factors, such as the mode of delivery, diet, the environment in which the child grows, and the use of various medications, particularly antibiotics. Understanding the factors that contribute to dysbiosis can aid in developing strategies to promote a healthy microbiome in children, which is essential for preventing various diseases later in life (3). Altered function and composition of gut microbiota during early childhood can lead to a variety of health issues. The improper balance of gut microbiota may disrupt the maturation of the immune system. This increases the likelihood of an exaggerated Th2 immune response, characterized by elevated production of specific molecules such as interleukins (IL-4, IL-5, IL-13) and IgE antibodies, which are key players in the development of allergic diseases, including asthma, eczema, and food allergies (4). Additionally, early-life dysbiosis is associated with an increased risk of metabolic diseases such as diabetes and obesity, as well as a higher susceptibility to developing inflammatory bowel diseases during adolescence (3).

Thus, in children whose microbiota is still developing and maturing, the early establishment of a healthy gut microbiome is linked to long-term health outcomes and the introduction of probiotics may offer protective effects against various disorders. Research indicates that probiotics may help prevent and manage common pediatric health conditions such as diarrhea, constipation, and colic. Moreover, emerging evidence suggests that the positive effects of probiotics extend beyond gastrointestinal health, including enhanced immune function, reduced inflammatory processes, mitigation of allergic reactions, improved skin health, regulation of metabolic disorders such as obesity and type 2 diabetes, and even support for mental well-being through the gut-brain axis (5–7).

Probiotics exert their effects through various mechanisms. These mechanisms include modulation of the gut microbiota composition by promoting the growth of other beneficial bacteria and enhancing the production of their metabolic byproducts. They also compete with pathogenic microorganisms for adhesion sites and nutrients, helping to prevent their colonization. Additionally, probiotics strengthen the intestinal barrier by reducing permeability, which in turn lowers inflammation and the risk of allergic reactions (8). Probiotics achieve many of their beneficial effects through the production of bioactive metabolites, such as short-chain fatty acids (SCFAs), bacteriocins, exopolysaccharides, vitamins, and neuroactive substances like gamma-aminobutyric acid (GABA) which play critical roles in their functionality. SCFAs such as acetate, propionate, and butyrate help maintain the gut’s protective barrier, enhance immune function, and provide energy to intestinal cells. These processes are particularly important in infants and young children, whose immune and digestive systems are still developing (9). Additionally, probiotics play a significant role in the absorption of essential nutrients in children. A recently published study has shown that these beneficial microorganisms can increase serum concentrations of vital vitamins such as vitamin D and A, along with minerals like calcium and zinc, thereby improving nutritional status and potentially boosting immunity (10). Probiotics can enhance respiratory, digestive, and immune functions due to their ability to promote humoral immunity maturation, particularly by increasing IgA antibody production (4).

What is important to know is that the effectiveness of probiotics can vary significantly based on individual health conditions and the specific strains used (9). This variability highlights the importance of personalized approaches to probiotic supplementation; what benefits one individual may not necessarily extend to another due to differences in gut microbiota composition or underlying health issues (11).

Of particular importance, yet often overlooked and insufficiently explored, is the fact that probiotics may interact with other drugs, influencing their absorption, metabolism, and effectiveness. Additionally, probiotics may modulate the activity of drug-metabolizing enzymes and transporters, thereby altering drug disposition (12–15). Understanding these interactions is vital for optimizing therapeutic outcomes, as inappropriate combinations can lead to reduced drug efficacy or an increased risk of adverse effects. Therefore, in addition to probiotic benefits it is essential to carefully evaluate the use of probiotics alongside medications, underscoring the importance of personalized approaches to treatment.

Since the effectiveness of probiotics depends on various factors, including the selection of the appropriate probiotic strain, the correct dose, and the duration of use, parental education about probiotics is of utmost importance. Well-informed parents are more likely to understand the benefits and limitations of probiotic therapy, which may lead to its appropriate use and maximize its potential health benefits for their children. Several studies have shown that parents’ awareness and practices regarding probiotics vary widely across different countries, with significant gaps in understanding (16–18).

To the best of our knowledge, no studies on this topic have been conducted in Serbia thus far. The only existing study, our previous research, assessed the knowledge, attitudes, and practices of health science students regarding gut microbiota and probiotics, revealing significant knowledge gaps and deficiencies in their practical application (19). Therefore, the aim of this study was to assess the knowledge, attitudes, and practices of parents regarding the use of probiotics in preschool-aged children, as well as to identify factors associated with probiotic supplementation. Additionally, the goal was to identify key factors that influence parents’ knowledge and attitudes toward probiotics and to guide future educational efforts aimed at enhancing awareness and promoting proper probiotic use.

## 2 Methodology

### 2.1 Study design

The study was conducted as a cross-sectional study in Serbia, following approval from the Ethics Committee of the Faculty of Medicine, University of Novi Sad (No 01-39/278). It was carried out from November 2024 to January 2025 through an anonymous survey in the form of a questionnaire assessing parents’ awareness of probiotics and their use in children. The participants were parents of preschool-aged children (1–7 years). To estimate the number of children aged 1–7 years in Serbia, we used the official data on the total population of approximately 6.7 million people and the average natality rate of 9–10 births per 1,000 inhabitants (stat.gov.rs). Based on this data, the annual number of births in Serbia is estimated to be between 60,300 and 67,000. Assuming an even distribution of births across all age groups, the number of children aged 1–7 years (covering 7 age groups) is estimated to be between 420,000 and 470,000. Based on this estimation, the minimum sample size for the study, with a 95% confidence level, a 5% margin of error, and a 50% response distribution, was calculated to be around 385 participants.

### 2.2 Questionnaire

The questionnaire was created in electronic form using Google Forms and distributed to parents through informal groups on social media, with a total of 1,625 parents completing it.

The questionnaire for this study was developed by combining questions from previously conducted surveys (16, 18, 19), with modifications to ensure improved clarity for non-medical respondents and better reflection of the specific needs and practices of parents, such as their reasons for administering probiotics and the duration of use, and the sources of information they rely on. It also included new questions specifically designed to enhance the sections assessing parental knowledge and attitudes, allowing for a more comprehensive evaluation of these aspects. Furthermore, questions addressing interactions between probiotics and other medications were incorporated, as this important aspect was not previously covered in similar questionnaires distributed to parents, thereby contributing to a more comprehensive understanding of parental knowledge and practices. The content, readability, comprehension, and design of the questionnaire were pretested on 15 parents, 5 pediatricians, and 10 professors from the Faculty of Medicine in Novi Sad. Based on their feedback, certain questions and response options were refined to enhance clarity and usability.

Detailed information about the survey was provided on the first page of the questionnaire. Before completing the questionnaire, all participants gave informed consent. No incentives or compensation were provided for participation in the study, in order to minimize response bias related to social desirability or external motivation. The questionnaire was divided into four sections. The first section focused on the socio-demographic characteristics of the parents (gender, age, education level, employment status, place of residence), as well as key information related to the child for whom the parent completed the questionnaire (gender, age, health and immunity status of the child, use of other medications and supplements, etc.). The second section explored parents’ experiences with probiotic use in children. Questions in this section focused on the situations in which parents usually administered probiotics, the longest duration of continuous probiotic use, and whether they consulted a physician or pediatrician before administration. Parents were also asked how they selected probiotics (e.g., based on medical or pharmaceutical advice, composition, price, or brand), and which probiotic strains or combinations they most commonly used. Additional questions assessed whether parents had observed any side effects, whether probiotics were given alongside other medications (excluding antibiotics), and if so, whether they were administered concurrently or separately.

The third section of the questionnaire assessed the parents’ knowledge of probiotics and consisted of 10 statements. Parents were asked to indicate whether they thought a given statement was true or false, with an option to indicate if they were unsure. Based on the total number of correct answers (ranging from 0 to 10), actual (objective) knowledge was categorized as good (>75%, 8–10 correct answers), fair (50–75%, 5–7 correct answers), and poor (<50%, 0–4 correct answers). These cut-offs were based on thresholds commonly used in in knowledge, attitudes, and practices studies related to health topics (19, 20). In addition to the actual knowledge score, calculated as the number of correct answers in the knowledge section, participants were also asked to rate their own knowledge about probiotics using a five-point Likert scale ranging from 1 (“very poor”) to 5 (“very good”), yielding a self-perceived knowledge score. To facilitate comparison with the actual knowledge scores (ranging from 0 to 10), both self-perceived and actual knowledge scores were normalized to a 0–100% scale. This approach allowed for visual comparison and correlation analysis between perceived and measured knowledge levels. The fourth and final section assessed parents’ attitudes using a Likert scale (1-5), where participants indicated their degree of agreement with seven statements. For statements reflecting a positive attitude toward probiotics, a higher score (5 – strongly agree) indicated a more positive attitude, while for statements expressing skepticism or a negative attitude (e.g., “I doubt the effectiveness of probiotics”), the scoring was reversed, so a higher degree of agreement received a lower score, and disagreement received a higher score (5 – strongly disagree). Based on the obtained values, an overall attitude score (ranging from 7 to 35) was calculated and ranked similarly to the knowledge score. ​Scores above 75% of the maximum score indicated a positive attitude toward probiotic use. A moderate score, ranging between 50-75% points, reflected a fair attitude, while scores below 50% indicated a poor attitude. A complete version of the questionnaire is included as a **Supplementary Material**.

### 2.3 Data analysis

After the study was completed, the data were exported in CSV format, imported into Excel, and further processed using standard statistical methods in IBM SPSS software (version 22.0, SPSS Inc., Chicago, IL, USA). Descriptive statistics were used to summarize numerical data, including the calculation of mean, median, minimum, maximum, and standard deviation. Categorical data were presented through percentages and frequencies. The Kolmogorov-Smirnov test was used to assess the normality of data distribution. Since the assumption of normal distribution was not met, the non-parametric Mann-Whitney U test was applied for comparing two independent samples, while the Kruskal-Wallis test was used for more than three samples. The Chi-square (χ²) test of independence was used to examine the relationship between categorical variables, and Spearman’s correlation coefficient was applied to assess the correlation between variables that did not follow a normal distribution. Statistical hypotheses were tested at a significance level (alpha level) of 0.05.

#### 2.3.1 Analysis of factors influencing the knowledge score and attitude score on probiotics

Binary logistic regression models were employed to identify factors associated with adequate knowledge and positive attitudes toward probiotics. The general form of the binary logistic regression model is:


$$logit\left(P\right)=ln(\frac{P}{1-P})=\beta_{0}+\beta_{1}X_{1}+\beta_{2}X_{2}+\ldots +\beta_{n}X_{n}$$

Where:

-
P is the probability of the event occurring (e.g., having good knowledge or a positive attitude)
-
$\frac{\text{P}}{1-\text{P}}$ is the odds of the event occurring versus not occurring
-
$\text{ln}\left(\frac{\text{P}}{1-\text{P}}\right)\;$ is the natural logarithm of odds (ln-odds or logit)
-
${\text{β}}_{0}$ is the intercept, the log-odds of the event when all predictors are zero
-
${\text{β}}_{1},{\text{ β}}_{2},\ldots ,{\text{β}}_{\text{n}}$ are the regression coefficients, representing the change in log-odds for each one-unit change in the corresponding independent variable
-
${\text{X}}_{1},{\text{ X}}_{2},\ldots ,{\text{X}}_{\text{n}}$ are the independent variables (predictors), such as parental age, education level, occupation in healthcare, etc.

Two separate models were developed for each dependent variable: “Good Knowledge” (yes/no) and “Positive Attitude” (yes/no). Good knowledge and positive attitude were defined as scores above 75%. The independent variables included in the models were: parental age (below 31/31-35/over 35), education level (under university degree/university degree/above university degree), number of children in the family (less than 2/2/more than 2), occupation related to health care (yes/no), duration of probiotic use (less than 10 days/more than 10 days), and whether the parent pays attention to the choice of probiotic strain (yes/no). The logistic regression model was constructed using a stepwise selection approach, wherein variables were retained based on their statistical significance (p < 0.05) and their contribution to the model’s overall goodness of fit. Multicollinearity was assessed using the variance inflation factor (VIF). All VIF values were below 2, indicating no significant multicollinearity among the predictors. The results are presented as odds ratios (ORs) with corresponding 95% confidence intervals. A p-value of less than 0.05 was considered statistically significant for all tests.

## 3 Results

### 3.1 Sociodemographic characteristics of parents and children

**Table 1** presents the basic sociodemographic characteristics of the parents and children for whom the questionnaire was completed. A total of 1,625 parents participated in the study, with the majority being mothers (98.6%). The largest group of parents falls within the age range of 31 to 35 years (41%). In terms of education, over half of the respondents (56.9%) hold a university degree. Most participants reside in urban areas (78.3%), while 11% live in rural regions and 10.6% in suburban settlements. Regarding employment status, the majority of parents are employed (80.4%). Professionally, nearly a quarter of respondents (24.2%) have occupation related to healthcare. Among the 1,625 children, boys slightly outnumber girls (52.5% vs. 47.5%). The children are aged 1–7 years, with a gradual decrease in the number of respondents in older age groups.

**Table 1:** Sociodemographic characteristics of the parents and child.

Variable	Frequency (n)	Percentage (%)
Parent		
Gender		
Female	1603	98.6
Male	22	1.4
Age		
18-25	59	3.6
26-30	343	21.1
31-35	667	41.0
36-40	441	27.1
41-45	98	6.0
Over 45	17	1.0
Education level		
Primary school	3	0.2
High school	346	21.3
Higher vocational education (non-university degree)	260	16.0
Bachelor’s or master’s degree (university degree)	925	56.9
Postgraduate education (PhD, specialization)	91	5.6
Place of residence		
Urban area	1273	78.3
Rural area	179	11.0
Suburban area	173	10.6
Employment status		
Employed	1307	80.4
Unemployed	300	18.5
Student	18	1.1
Healtdcare professional		
No	1231	75.8
Yes	394	24.2
Number of children in family		
1	678	41.7
2	743	45.7
3	186	11.4
4	15	0.9
5 or more	3	0.2
Child		
Gender		
Female	772	47.5
Male	853	52.5
Age		
1	314	19.3
2	310	19.1
3	273	16.8
4	256	15.8
5	188	11.6
6	166	10.2
7	118	7.3
TOTAL	1625	100

### 3.2 Health problems, medications, and supplement use in the past year

**Figure 1:**
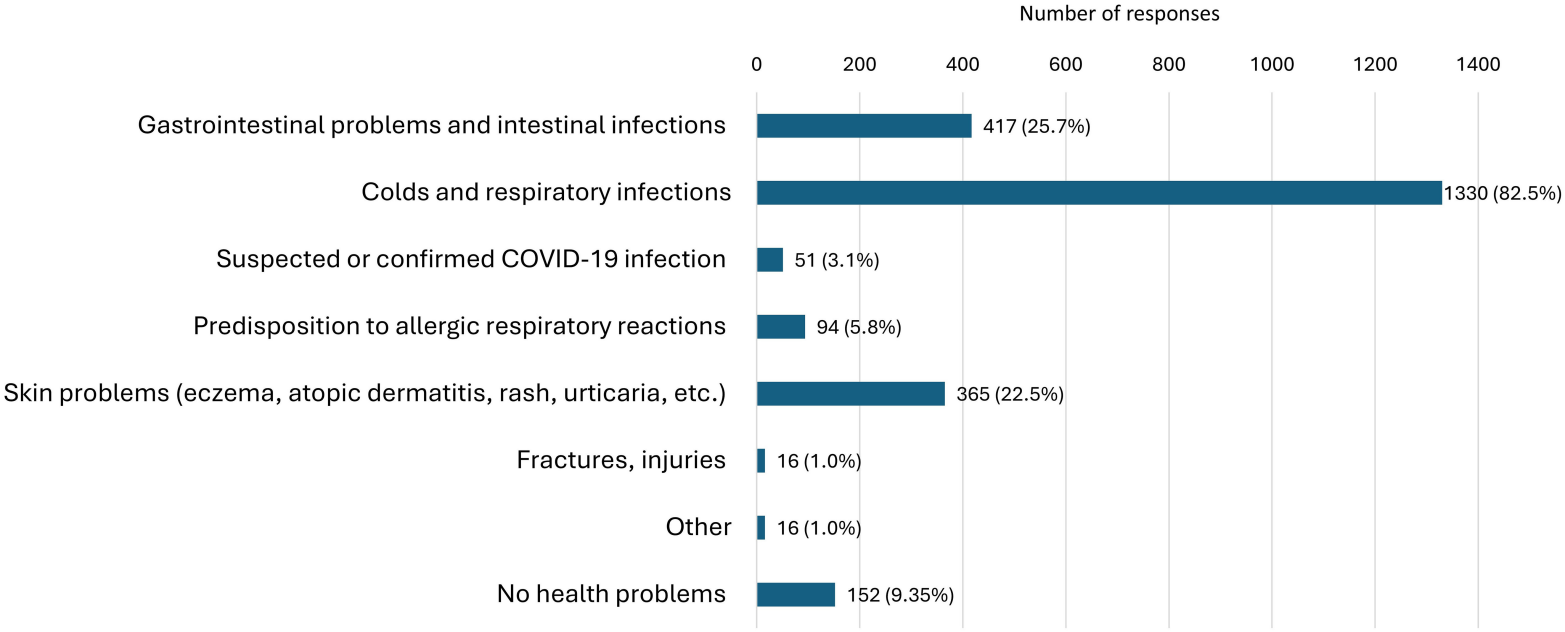
Parent-reported health conditions in children in the past year.

The major reported health issues in children over the past year were colds and other respiratory infections, reported in 82.5% of cases (**Figure 1**). Additionally, a significant proportion of children (25.7%) experienced gastrointestinal issues. Apart from these complaints, skin problems were observed in 22.5% of children, while 5.8% of respondents reported a predisposition to allergic respiratory reactions. Suspected or confirmed cases of COVID-19 infection in children were reported by 3.1% of parents. Almost 10% of parents stated that their children had no health issues in the past year.

**Figure 2:**
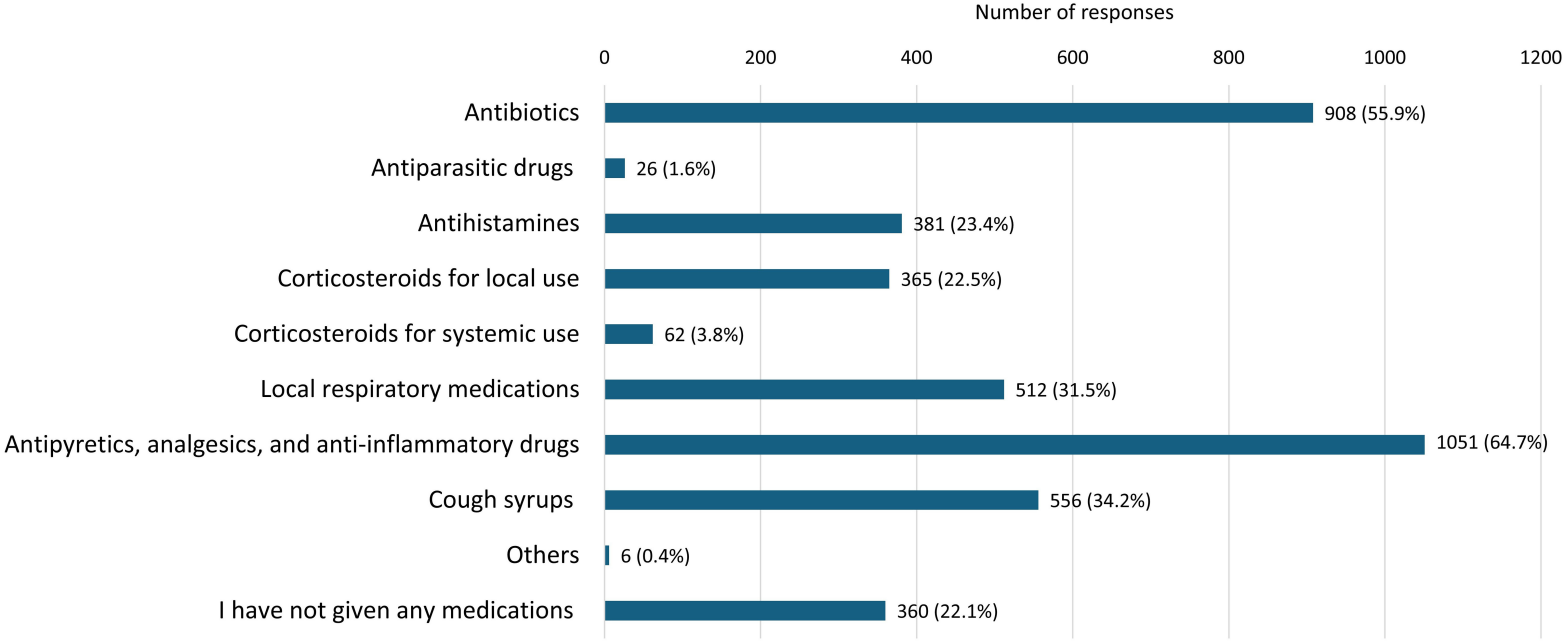
Medications used by the child in the past 12 months.

Data on the medications used by children in the past 12 months (**Figure 2**) show that analgoantipyretics and antibiotics were the most commonly used, with 64.7% and 55.9% of respondents reporting their use, respectively. In addition, a significant percentage of children (34.2%) used cough medications. Antihistamines were used by 23.4% of children, while local corticosteroids in the form of inhalers, creams, or ointments were used by 22.5% of children. Local medications for respiratory issues, including inhalers and nasal drops, were used by 31.5% of children. Less frequently, systemic corticosteroids in the form of injections or tablets (3.8%) and antiparasitic drugs (1.6%) and were reported. Notably, 22.1% of respondents reported that they did not administer any medications to their children during the past 12 months.

**Figure 3:**
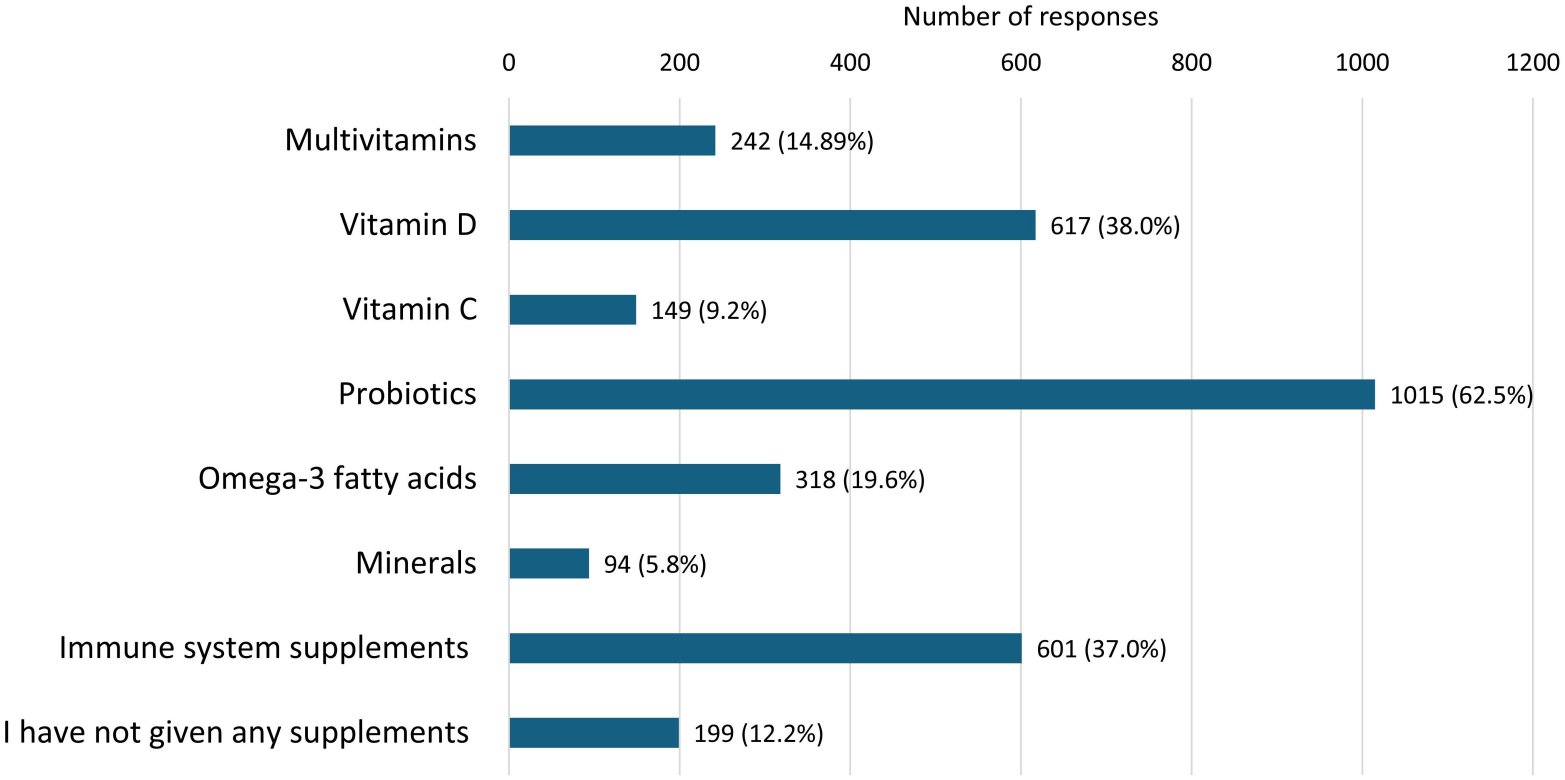
Supplements used by the child in the past 12 months.

Data on supplement use in children over the past year (**Figure 3**) show that probiotics were the most frequently used, with 62.4% of participants reporting their use. Vitamin D was also highly prevalent, with 38.0% of children using it. Immune-boosting supplements were reported by 37.0% of respondents, while multivitamin supplements were administered to 14.9% of children. Omega-3 fatty acids were used by 19.6% of children, while vitamin C and minerals such as iron, calcium, magnesium, and zinc were less commonly used, with prevalence rates of 9.2% and 5.8%, respectively. A certain percentage of parents (12.2%) reported not giving any supplements to their children.

### 3.3 Bivariate analysis of factors associated with probiotic use in children

#### 3.3.1 Association between probiotic use and health issues in children, as well as medication use in the past year

Analysis of data from **Table 2**, obtained using the Pearson’s χ² test of independence, reveals a statistically significant association between probiotic use and the presence of certain health conditions in children over the past year, as well as with medication use during the same period. In the specified period, 62.5% of children used probiotics, while 37.5% did not. Children who used probiotics showed a slightly higher incidence of gastrointestinal issues (29.3% vs. 19.7%), with a weak positive correlation observed (φ = 0.106, p < 0.001). Additionally, colds and other respiratory infections were more frequent among children who consumed probiotics (84.8% vs. 76.9%), with a weak positive correlation (φ = 0.100, p < 0.001). Children using probiotics also exhibited a higher incidence of a predisposition to allergic respiratory reactions (6.7% vs. 4.3%), although the correlation was very weak (φ = 0.051, p = 0.042). Similarly, skin problems were somewhat more common in this group (24.5% vs. 19.0%), with a weak correlation (φ = 0.064, p = 0.010). On the other hand, there was no significant difference in the incidence of COVID-19 between the groups. Children who did not use probiotics were more likely to report no health issues (14.3% vs. 6.4%), with a weak negative correlation (φ = -0.131, p < 0.001), suggesting that health issues were more common in probiotic users. Furthermore, children who used probiotics were more likely to have used medication in the past year compared to those who did not use probiotics (80.8% vs. 70.8%), with a weak positive correlation (φ = 0.115, p < 0.001). These results suggest that probiotic use was associated with a slightly higher frequency of health issues and more frequent medication use, although all the observed correlations were of weak intensity.

**Table 2:** Bivariate analysis of the association between probiotic use, child health issues, and medication use in the past year, using Chi-square tests.

	Total n (%)	Probiotic users n (%)	Non-Probiotic users n (%)	value	df	φ	p
Health issues in the past 12 months							
Gastrointestinal problems							
Yes	417 (25.7%)	297 (29.3%)	120 (19.7%)	18.365	1	0.106	0.000*
No	1208 (74.3%)	718 (70.7%)	490 (80.3%)				
Colds and respiratory infections							
Yes	1330 (81.8%)	861 (84.8%)	469 (76.9%)	16.176	1	0.100	0.000*
No	295 (18.2%)	154 (15.2%)	141 (23.1%)				
Allergic respiratory reactions							
Yes	94 (5.8%)	68 (6.7%)	26 (4.3%)	4.153	1	0.051	0.042*
No	1531 (94.2%)	947 (93.3%)	584 (95.7%)				
Skin problems (eczema, atopic dermatitis, rash, urticaria, etc.)							
Yes	365 (22.5%)	249 (24.5%)	116 (19%)	6.665	1	0.064	0.010*
No	1260 (77.5%)	766 (75.5%)	494 (81.0%)				
COVID 19							
Yes	51 (3.1%)	35 (3.4%)	16 (2.6%)	0.854	1	0.023	0.355
No	1574 (96.9%)	980 (96.6%)	594 (97.4%)				
No health problems							
Yes	152 (9.4%)	65 (6.4%)	87 (14.3%)	27.750	1	-0.131	0.000*
No	1473 (90.6%)	950 (93.6%)	523 (85.7%)				
Medications used in the past 12 months							
Yes	1252 (77.0%)	820 (80.8%)	432 (70.8%)	21.409	1	0.115	0.000*
No	373 (23.0%)	195 (19.2%)	178 (29.2%)				
Total	1625	1015 (62.5%)	610 (37.5%)				

*Denote the p-values < 0.05.

#### 3.3.2 Association between probiotic use and child’s age, immunity score, parental knowledge, and attitudes

To examine the relationship between probiotic use and factors such as child’s age, immunity score, and parents’ knowledge and attitudes, Spearman’s correlation coefficient (ρ) was used. Probiotic use showed a very weak but statistically significant negative correlation with the child’s age (ρ = -0.084, p = 0.001), indicating that younger children were slightly more likely to receive probiotics compared to older children. Additionally, a weak but significant positive correlation was observed between probiotic use and the parents’ knowledge score (ρ = 0.136, p = 0.000), with parents of children using probiotics achieving slightly higher scores on the knowledge test. Furthermore, a moderate positive correlation was found between probiotic use and the attitude score (ρ = 0.249, p = 0.000), suggesting that parents of children using probiotics had a more positive attitude toward probiotics compared to parents of children who did not use probiotics. In contrast, probiotic use showed a very weak negative correlation with the parents’ assessment of the child’s immunity (ρ = -0.078, p = 0.002), with parents of children who did not use probiotics rating their children’s immunity statistically significantly higher than those who administered probiotics to their children.

### 3.4 The overall knowledge and attitude scores of parents regarding probiotics and comparison based on probiotic use in children

**Figure 4:**
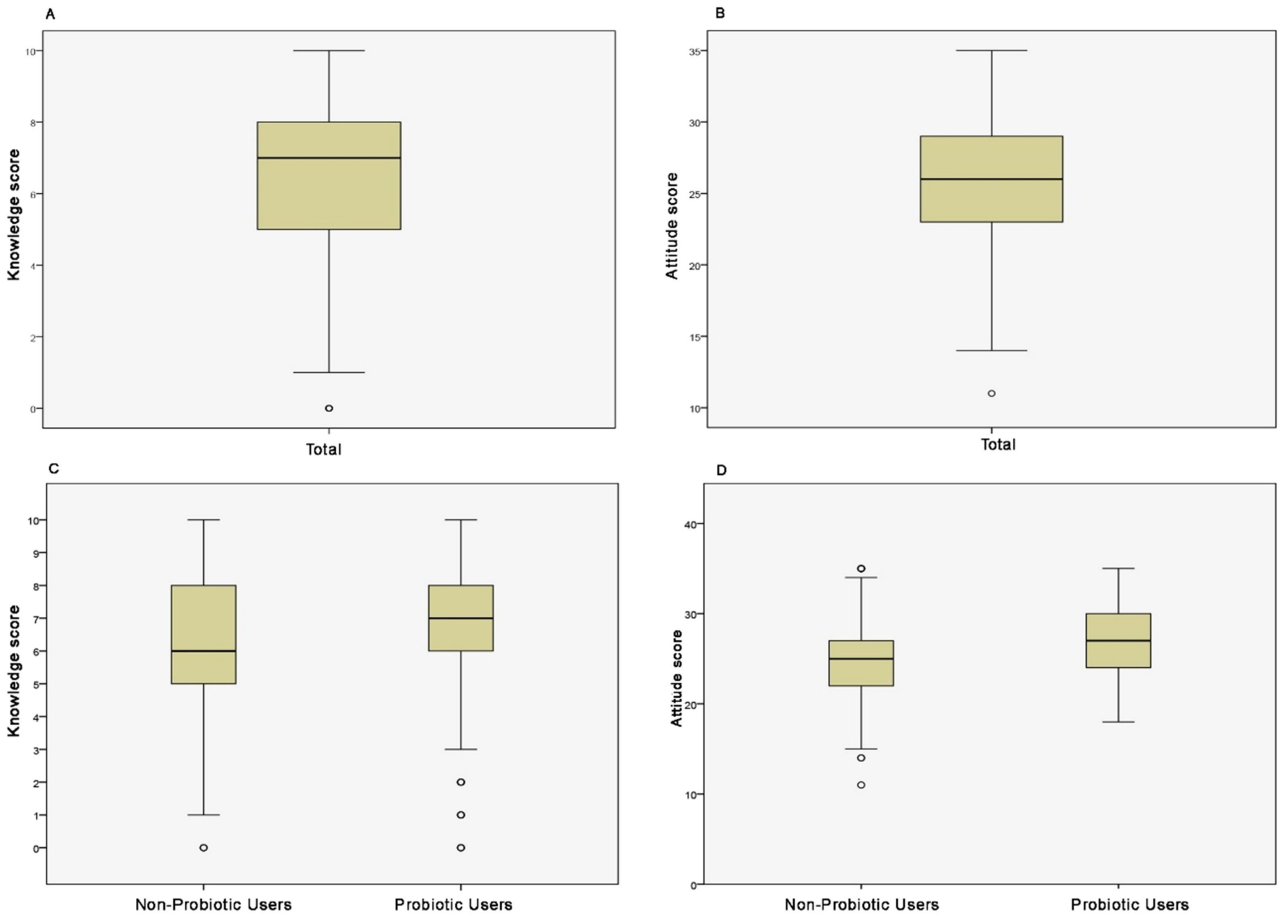
Knowledge and attitude scores of parents of all children **(A, B)**, as well as those whose children used and did not use probiotics **(C, D)**, respectively.

The median knowledge score of all respondents was 7.0 with an interquartile range (IQR) of 6–8, while the median attitude score was 26.0 (IQR: 23-29) on their respective scales (0–10 for knowledge and 5–35 for attitude). When comparing groups based on probiotic use, among respondents who did not use probiotics, the median knowledge score was 6.0 (IQR: 4–7), while the median attitude score was 25.0 (IQR: 22–27). In contrast, in the probiotic group, the median knowledge score was 7.0 (IQR: 6–8), and the median attitude score was 27.0 (IQR: 25–31). Mann–Whitney U test revealed statistically significant differences between the groups (p < 0.001), indicating that parents who administered probiotics had significantly higher knowledge and attitude scores. These results are illustrated in **Figure 4**. For descriptive purposes, the mean knowledge score of all respondents was 6.49 ± 1.81, and the mean attitude score was 26.18 ± 3.91. The mean knowledge score in the non-probiotic group was 6.16 ± 1.93, compared to 6.68 ± 1.71 in the probiotic group, while the mean attitude score was 24.91 ± 3.91 and 26.94 ± 3.71, respectively.

**Figure 5:**
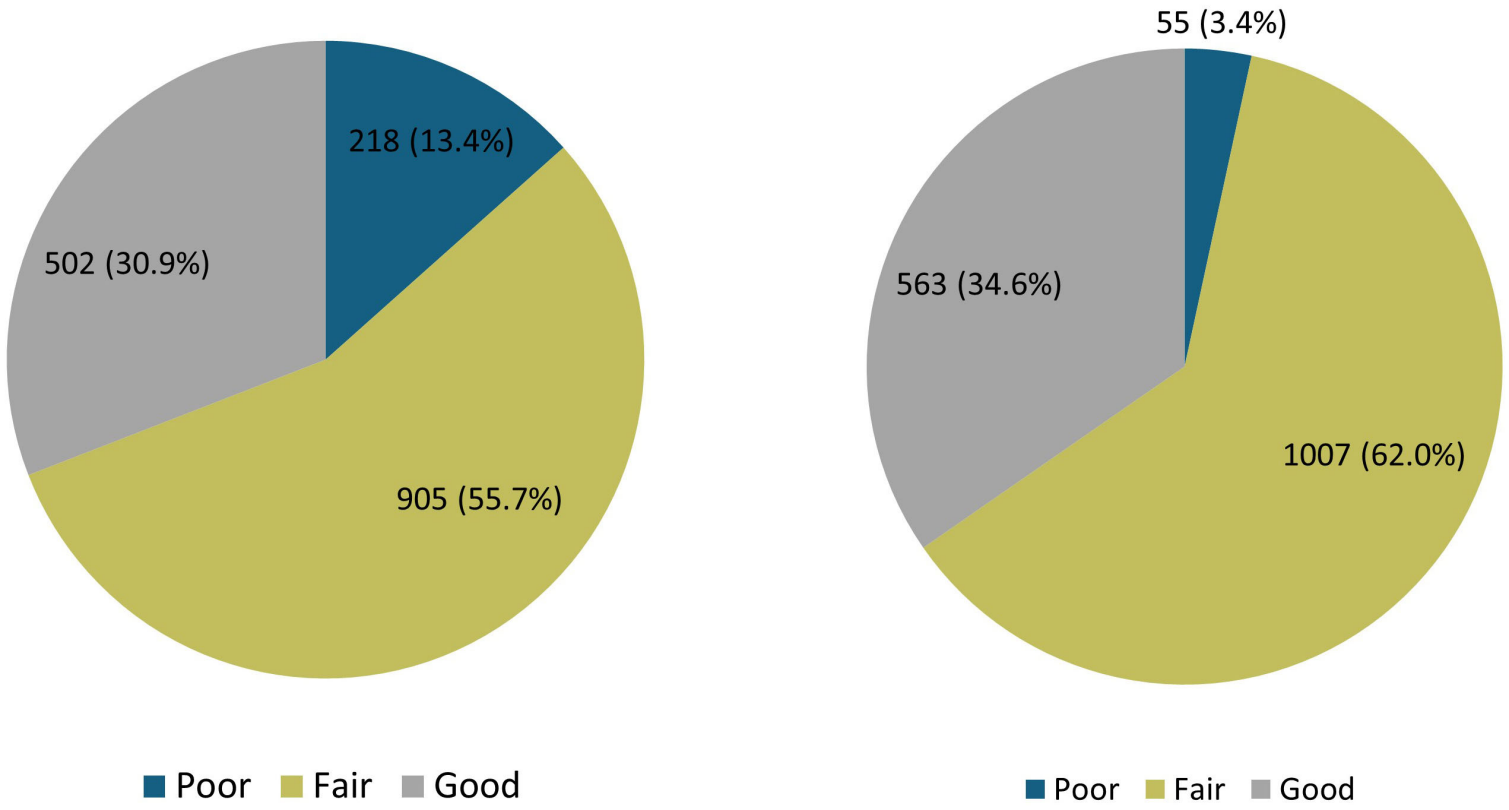
Distribution of knowledge and attitude scores among respondents.

**Figure 5** illustrates the distribution of knowledge and attitude scores among respondents. The knowledge distribution showed that only 30.9% of respondents demonstrated good knowledge, while the largest proportion, 55.7% of respondents, demonstrated fair knowledge, and 13.4% exhibited poor knowledge. Similarly, the distribution of attitudes indicated that the majority of respondents (62.0%) had a fair attitude score. A positive attitude toward probiotics was expressed by 34.6% of respondents, while 3.4% had a poor attitude score.

### 3.5 Self-perceived knowledge

**Figure 6:**
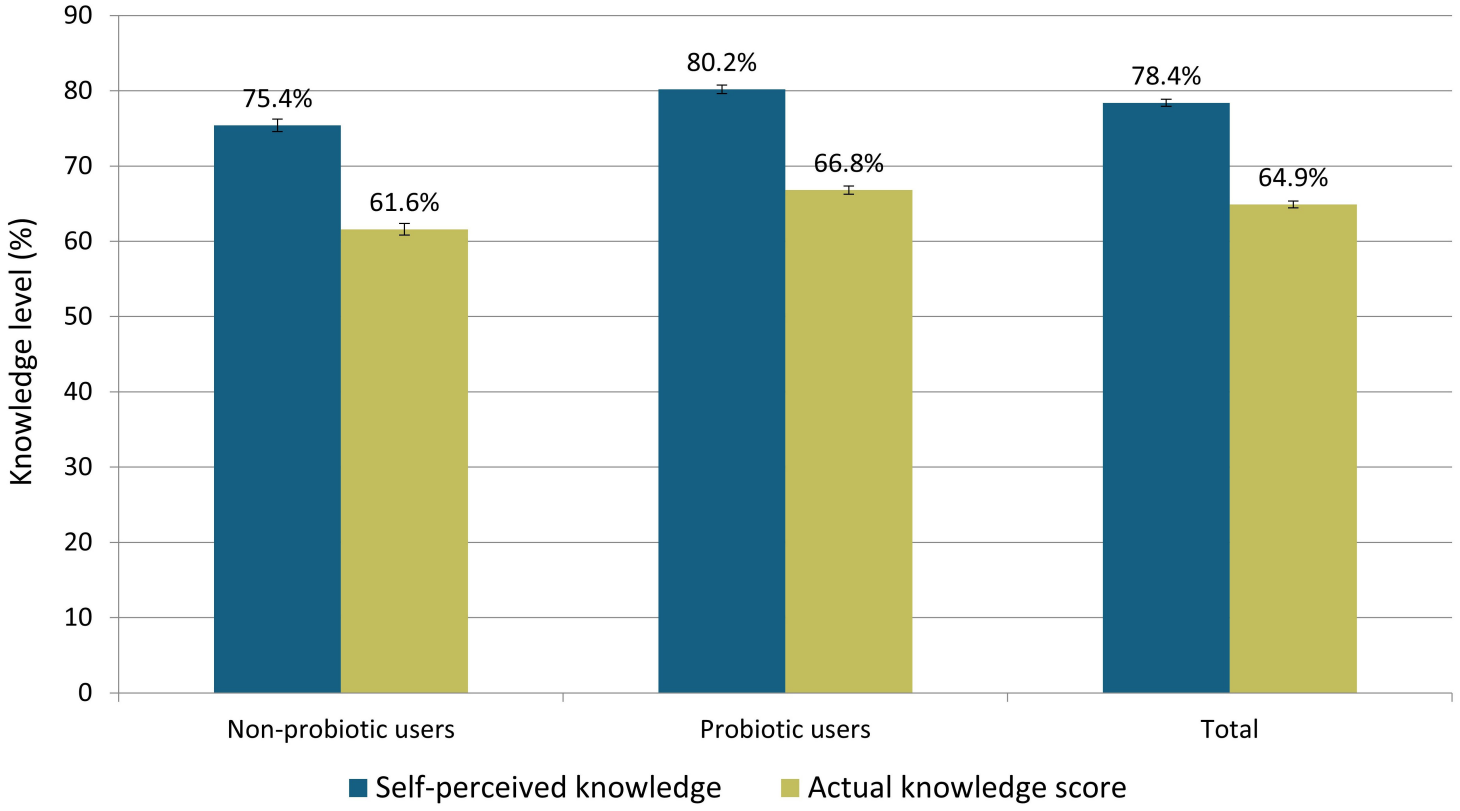
Comparison of self-perceived and actual knowledge about probiotics between groups that did not use and did use probiotics, and total.

Based on the Mann-Whitney U test, a statistically significant, but weak to moderate positive correlation was found between the level of self-perceived knowledge about probiotics and the actual knowledge score (ρ = 0.307, p = 0.000) (**Figure 6** **).** Additionally, it was observed that in the group of respondents who used probiotics, both the self-perceived knowledge level and the actual knowledge score were higher compared to the group that did not use probiotics.

### 3.6 Parental knowledge of probiotics

**Figure 7:**
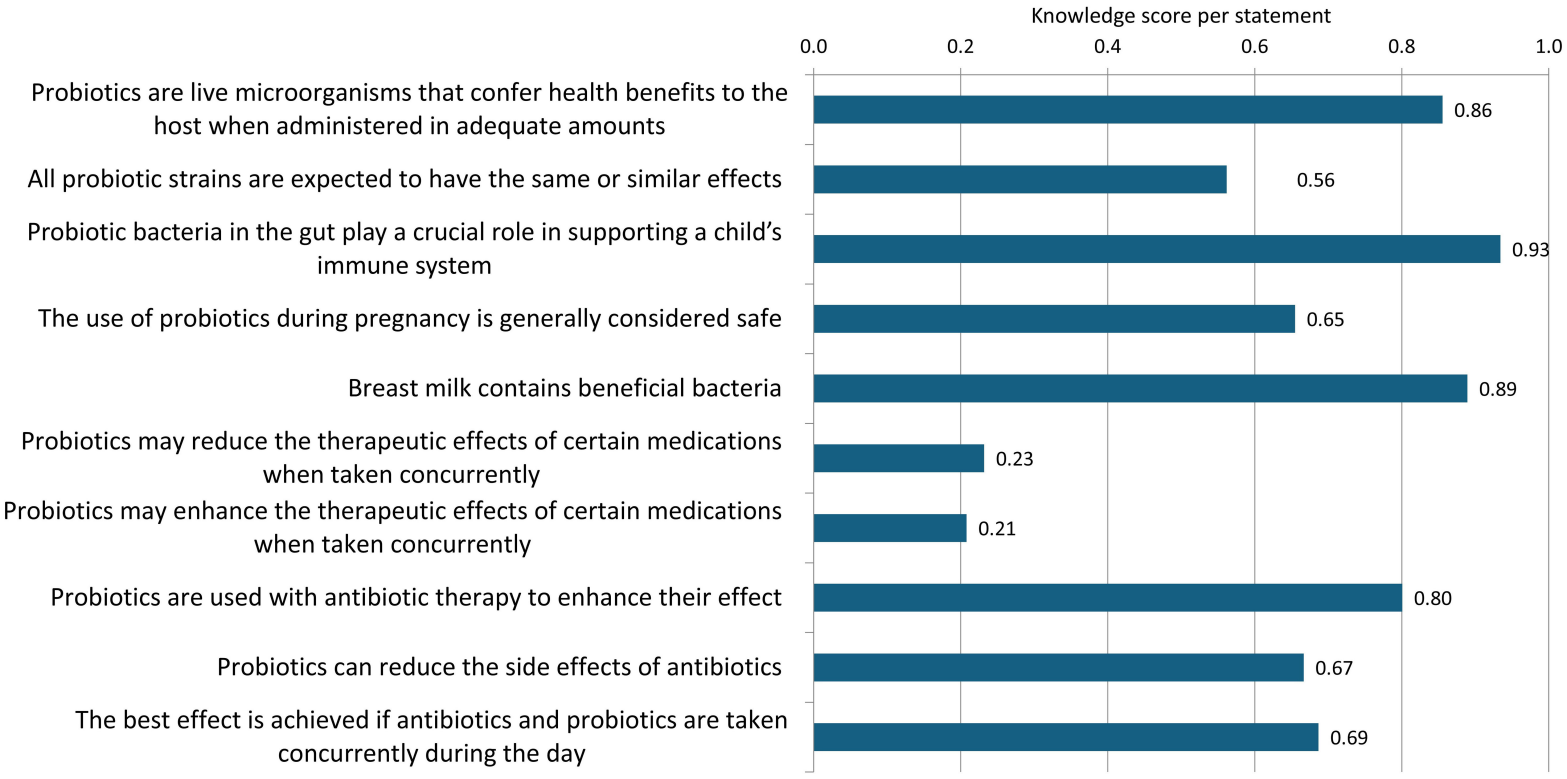
Mean knowledge scores per statement.

**Figure 7** presents the mean knowledge score of parents for each statement regarding probiotics, where correct answers are scored as 1 and incorrect or unsure answers are scored as 0.

Parents demonstrated a high level of knowledge about the basic characteristics of probiotics, including the importance of probiotic bacteria for child immunity (score 0.93), the presence of beneficial bacteria in breast milk (score 0.89), and understanding the definition of probiotics (score 0.86). There was insufficient knowledge regarding the safety of probiotic use during pregnancy, with approximately 35% of respondents unsure or believing it is unsafe to use probiotics during this period. Additionally, there was some uncertainty about the specificity of probiotic strains, with just over half of the respondents aware that not all probiotic strains can be expected to produce the same effects (score 0.56). Parents generally understood the rationale for using probiotics during antibiotic therapy to reduce the adverse effects of antibiotics (score 0.67), but not for enhancing the effect of antibiotics (score 0.8). Around 70% of respondents were aware that probiotics should not be taken simultaneously with antibiotics to achieve the desired effect. The weakest knowledge was observed regarding the potential interactions between probiotics and medications, both in terms of the possibility of probiotics reducing the effect of other medications when taken together (score 0.23) and in terms of enhancing the effect of certain medications (score 0.21).

### 3.7 Parental attitudes toward probiotics

**Figure 8:**
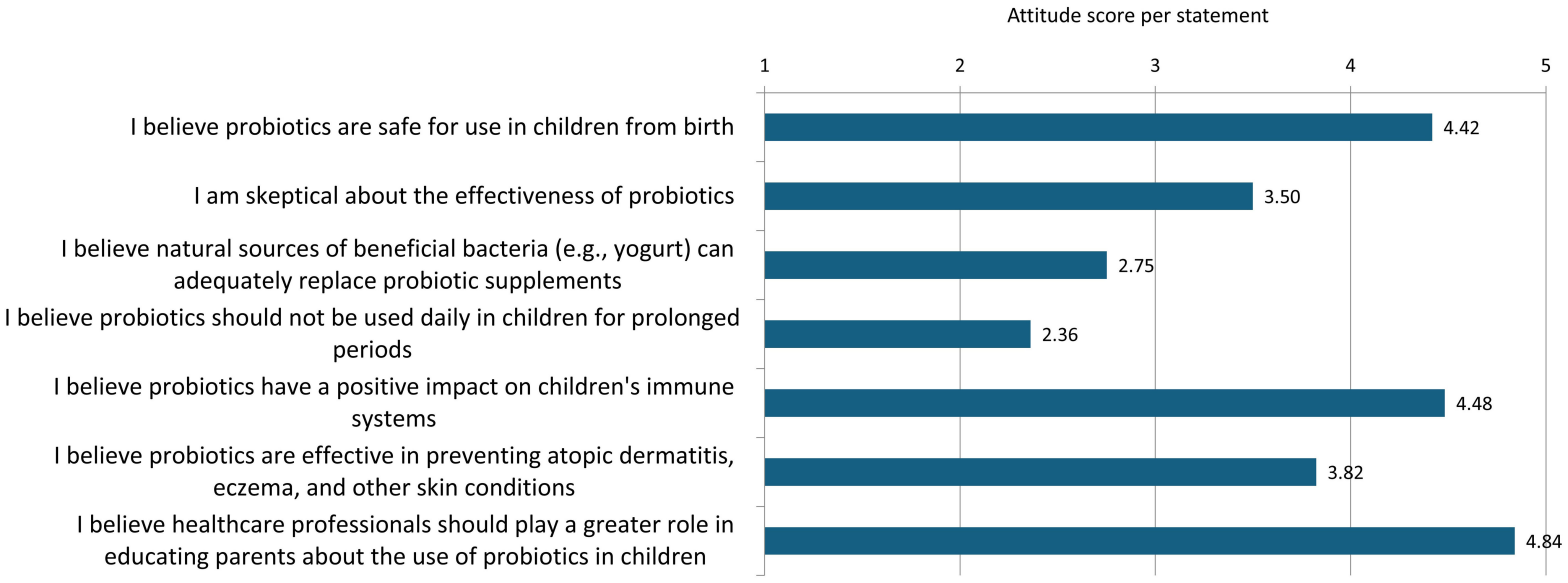
Mean attitude scores per statement.

The results show that the mean ratings of parents’ attitudes toward probiotics varied between 2.36 and 4.84 on a scale from 1 to 5 (**Figure 8**).

The highest mean rating (4.84) was given to the statement that healthcare professionals should be more engaged in informing parents about the use of probiotics in children, a view supported by over 90% of respondents. The belief that probiotics have a positive effect on children’s immune systems also received a high rating (4.48), with about 85% of respondents agreeing with this statement. Over 80% of parents agreed that probiotic use in children is safe from birth (4.42). A moderate level of agreement (3.82) was observed for the statement that probiotics may have an effect on the prevention of atopic dermatitis, eczema, and other skin problems, with 41.7% of respondents expressing uncertainty regarding this claim. About half of the parents expressed doubts about the effectiveness of probiotics (score 3.50). Similarly, nearly half of the respondents (47.8%) believed that natural sources of bacteria are an adequate substitute for probiotic supplements (score 2.75). The attitude toward the duration of probiotic use received the lowest rating (2.36), with almost 70% of parents believing that children should not take probiotics daily for long periods, while 23% of respondents were uncertain about the recommended duration of use.

### 3.8 Previous experience with probiotic use

Parental information on probiotic use is presented in **Table 3**. The questions regarding the use of probiotics in children were answered only by those parents who reported that they had given probiotics to their child at some point, which included 1,575 respondents, or 95.7% of the total sample.

**Table 3:** Parental experience with probiotic use in children.

Variable	Frequency (n)	Percentage (%)
In what situations do you usually give probiotics to your child?		
During antibiotic therapy	1184	75.2
Gastrointestinal issues (diarrhea, constipation, nausea, vomiting, intestinal infections, etc.)	1091	69.3
Respiratory issues (cold, cough, runny nose, pneumonia, etc.)	85	5.4
Prevention and strengthening immunity	717	45.5
Prevention and treatment of allergic reactions	125	7.9
Prevention and reduction of skin disease symptoms (atopic dermatitis, eczema, etc.)	172	10.9
Other	49	3.1
What is the longest period you have given probiotics to your child continuously?		
Less than 10 days	576	36.6
10–30 days	398	25.3
1–3 months	256	16.2
4–6 months	99	6.3
More than 6 months	243	15.4
No response	3	0.2
Do you consult with your chosen physician or pediatrician before giving probiotics to your child?		
Never	275	17.5
Sometimes	863	54.8
Always	437	27.7
How do you choose probiotics for your child?		
Based on doctor’s recommendation	894	56.8
Based on pharmacist’s recommendation	630	40.0
Based on the composition of the probiotic	492	31.2
Based on price	100	6.3
Based on brand/manufacturer	108	6.9
Other	17	1.1
Which probiotic composition do you most often give/have given to your child?		
Lactobacillus	478	30.3
Bifidobacterium	24	1.5
Saccharomyces boulardii	197	12.5
Combination of strains	407	25.8
I don’t pay attention	467	29.6
Other	2	0.1
Have you noticed any side effects while giving probiotics to your child?		
No	1543	98.0
Yes	32	2.0
Have you ever given probiotics to your child while taking other medications (other than antibiotics)?		
No	928	58.9
Yes	697	44.2
If you answered yes to the previous question, how did you administer the probiotic? (n=697)		
At the same time as the other medication (concurrently)	25	3.6
At a different time from the medication (separately)	636	91.2
I did not pay attention	36	5.2
TOTAL	1575	100%

Among those who gave probiotics to their child, 75.2% reported doing so during antibiotic therapy. The second most common reason was gastrointestinal problems such as diarrhea, constipation, and nausea (69.3%). Additionally, 45.5% of parents used probiotics for the prevention and strengthening of their child’s immune system. Probiotics were used by 10.92% of respondents to help prevent and alleviate symptoms of skin conditions, and an even smaller percentage (7.9%) used them for the prevention and treatment of allergic reactions. The smallest group of parents (5.4%) administered probiotics to their children in case of respiratory issues. In addition to the provided reasons, several parents selected the “Other” option, with a significant number mentioning travel as an additional reason for using probiotics.

Regarding the duration of probiotic use, the majority of parents (36.6%) administered probiotics to their children for fewer than ten days, while 15.4% of parents reported giving probiotics for a period exceeding six months. When asked whether they consult with their physician or pediatrician prior to administering probiotics to their child, 17.4% of parents indicated that they never seek medical advice, 54.8% consult occasionally, and 27.7% always obtain medical guidance. Responses to the inquiry about adverse effects during or after the administration of probiotics revealed that the vast majority of parents (98.0%) observed no negative effects, whereas only 2.0% reported adverse reactions, which included gastrointestinal issues such as constipation and diarrhea.

When it comes to the type of probiotics most frequently administered to their children, the most commonly used probiotic contains the *Lactobacillus* strain (30.3%), followed by *Bifidobacterium* and *Saccharomyces boulardii* in smaller proportions (1.5% and 12.5%, respectively). Combined strains are utilized by 25.8% of parents, while 29.6% do not consider the composition of the probiotics they use. In selecting a probiotic for their child, the majority of parents (56.8%) rely on their physician’s recommendation, while 40.0% seek advice from a pharmacist. The composition of the probiotic influences the decision of 31.2% of parents, whereas a smaller percentage choose probiotics based on price (6.3%) or brand/manufacturer (6.9%). In the “Other” category, 1.1% of parents indicated additional factors influencing their choice, with some noting that the formulation of the probiotic (e.g., powder, gummies, or liquid forms) preferred by the child is a significant factor.

Regarding the concurrent use of probiotics and other medications, 58.9% of parents reported that they did not administer probiotics alongside other medications, while 44.2% did. Among those who gave probiotics with other medications, 91.2% administered them separately, 5.16% did not pay attention to the timing, and 3.6% gave probiotics simultaneously with medications.

### 3.9 Predictive model of parental knowledge and attitude on toward probiotics

The binary logistic regression models identified significant predictors for both good knowledge and positive attitudes toward probiotics among parents. Both models were statistically significant as indicated by the Omnibus Test of Model Coefficients (χ² = 161.961, df = 9, p < 0.001 for knowledge; χ² = 206.833, df = 9, p < 0.001 for attitudes), indicating a strong association between the predictors and the outcomes. Additionally, the Hosmer-Lemeshow test showed no significant deviation for either model (χ² = 5.977, df = 8, p = 0.650 for knowledge; χ² = 9.248, df = 8, p = 0.322 for attitudes), confirming a good fit of the model to the data.

#### 3.9.1 Predictors of parental knowledge about probiotics

The results of the binary logistic regression analysis using the dichotomous variable ‘Good Knowledge of Drugs’ (yes/no) as the dependent factor and other independent variables mentioned above are shown in **Table 4**. The results indicate that parents without a university degree have approximately 37% lower odds of having adequate knowledge about probiotics compared to those with university education (OR = 0.633, p < 0.001), while parents with a postgraduate degree have approximately 88% higher odds of having adequate knowledge compared to those with a university education (OR = 1.876, p = 0.009). Additionally, healthcare professionals have nearly twice the odds of having adequate knowledge about probiotics compared to those whose occupation is not related to healthcare (OR = 1.874, p < 0.001). Parents who administer probiotics to their children for less than 10 days have approximately 53% lower odds of having adequate knowledge compared to those who use probiotics for longer periods (OR = 0.473, p < 0.001). Finally, parents who do not pay attention to the composition of probiotics have approximately 51% lower odds of having adequate knowledge compared to those who do (OR = 0.513, p < 0.001). The number of children in the family and parental age were not significant predictors of probiotic knowledge.

**Table 4:** Binary logistic regression analysis of factors associated with good knowledge about probiotics.

Independent variable	B	S.E.	Wald	df	p	OR	95% CI
Parental age							
Age below 31	-0.117	0.153	0.593	1	0.441	0.889	0.659 - 1.199
Age 31-35*	reference						
Age over 35	0.106	0.130	0.659	1	0.417	1.112	0.861 - 1.435
Education level							
Below university degree	-0.457	0.128	12.733	1	0.000	0.633	0.493 - 0.814
University degree*	reference						
Postgraduate degree	0.629	0.242	6.781	1	0.009	1.876	1.168 - 3.013
Healthcare professional							
No*	reference						
Yes	0.628	0.132	22.790	1	0.000	1.874	1.448 - 2.425
Number of children in family							
1	-0.035	0.122	0.080	1	0.777	0.966	0.761 - 1.227
2*	reference						
More than 2	-0.039	0.186	0.043	1	0.835	0.962	0.669 - 1.384
Duration of probiotic use							
Less than 10 days	-0.748	0.125	35.673	1	0.000	0.473	0.370 - 0.605
More than 10 days*	reference						
Paying attention to the choice of probiotic strain							
Yes*	reference						
No	-0.667	0.141	22.282	1	0.000	0.513	0.389 - 0.677
Constant	0.245	0.211	1.346	1	0.246	1.277	-

Tables description: Regression coefficients (B), standard errors (S.E.), Wald statistic, degrees of freedom (df), p-values, odds ratios (OR), and 95% confidence intervals (CI) for each independent variable. Significant predictors of good knowledge and positive parental attitudes toward probiotics are indicated by p-values < 0.05. *Denotes the reference category.

#### 3.9.2 Predictors of parental attitudes toward probiotic

**Table 5** presents the binary logistic regression results for predictors of positive parental attitudes toward probiotics. The findings suggest that parents without a university degree are about 25% less likely to have a positive attitude toward probiotics compared to those with a university education (OR = 0.747, p = 0.013). However, no significant difference in attitudes was observed between parents with postgraduate education and those with a university degree (OR = 1.229, p = 0.401). Healthcare professionals were more likely to have a positive attitude, with approximately 54% greater odds compared to non-healthcare professionals (OR = 1.542, p = 0.001). Parents who administered probiotics for less than 10 days had about 70% lower odds of holding a positive attitude compared to those who used them for longer periods (OR = 0.304, p < 0.001). Additionally, parents who did not consider probiotic composition when making a choice had about 48% lower odds of having a positive attitude compared to those who did (OR = 0.520, p < 0.001). Other examined factors, including parental age and the number of children in the household, did not show a statistically significant association with parental attitudes toward probiotics.

**Table 5:** Binary logistic regression analysis of factors associated with positive attitudes toward probiotics.

Independent variable	B	S.E.	Wald	df	p	OR	95% CI
Parental age							
Age below 31	0.065	0.140	0.215	1	0.643	1.067	0.811 - 1.406
Age 31-35*	reference						
Age over 35	-0.073	0.125	0.339	1	0.560	0.930	0.729 - 1.187
Education level							
Below university degree	-0.292	0.118	6.120	1	0.013	0.747	0.593 - 0.941
University degree*	reference						
Postgraduate degree	0.206	0.246	0.707	1	0.401	1.229	0.760 - 1.989
Healthcare professional							
No*	reference						
Yes	0.433	0.129	11.216	1	0.001	1.542	1.197 - 1.988
Number of children in family							
1	-0.079	0.115	0.467	1	0.494	0.924	0.737 - 1.158
2*	reference						
More than 2	0.062	0.173	0.127	1	0.722	1.064	0.758 - 1.493
Duration of probiotic use							
Less than 10 days	-1.191	0.115	107.214	1	0.000	0.304	0.243 - 0.381
More than 10 days*	reference						
Paying attention to the choice of probiotic strain							
Yes*	reference						
No	-0.654	0.125	27.293	1	0.000	0.520	0.407 - 0.665
Constant	1.053	0.196	28.929	1	0.000	2.866	-

Tables description: Regression coefficients (B), standard errors (S.E.), Wald statistic, degrees of freedom (df), p-values, odds ratios (OR), and 95% confidence intervals (CI) for each independent variable. Significant predictors of good knowledge and positive parental attitudes toward probiotics are indicated by p-values < 0.05. *Denotes the reference category.

## 4 Discussion

As insufficient parental awareness about probiotics may lead to the underuse of probiotics in appropriate clinical indications, improper strain selection, incorrect therapy duration, and neglect of potential drug interactions, all of which can compromise their efficacy and safety, this study aimed to examine the knowledge, attitudes, and previous experiences of parents regarding the use of probiotics in preschool-aged children in Serbia. To the best of our knowledge, this is the first study of this type conducted in Serbia. The high level of interest in this topic is reflected in the substantial response rate, with 1,625 respondents completing the questionnaire.

Since only 30.9% of respondents exhibited good knowledge about probiotics, the study highlights several crucial areas where parental understanding of probiotics could significantly influence their decisions regarding the use of these supplements. While parents generally understand basic concepts such as the definition of probiotics and their role in the immune system, their knowledge, attitudes, and practices remain limited in key areas, including strain-specific effects, appropriate duration of use, safety considerations, and potential interactions with medications.

An important aspect is the insufficient understanding of probiotic strain specificity, as clinical effects may vary depending on the target condition, which could lead to unrealistic expectations regarding their efficacy. While single-strain probiotics offer strain-specific and well-documented benefits, for example, *Lactobacillus rhamnosus* GG in preventing necrotizing enterocolitis, multi-strain formulations are often believed to provide broader health advantages due to potential synergistic interactions among different strains (21, 22). These synergistic effects may result in enhanced immune modulation, improved gut colonization, or broader antimicrobial action (23, 24). However, combining strains may also lead to antagonistic interactions, where one strain inhibits the growth or function of another, potentially reducing the overall efficacy or leading to unpredictable outcomes (25). Despite these theoretical risks, strong evidence of clinically relevant antagonism is currently limited. The decision to use single- or multi-strain probiotics should therefore be based on the specific health indication, available clinical evidence, and the safety profile of the selected strains.

One particularly concerning gap is the lack of knowledge regarding probiotic use during pregnancy, which suggests inadequate access to reliable information or the presence of conflicting opinions among healthcare professionals. It is essential to provide parents with clear guidelines on the safety and proper use of probiotics during this period. Studies have demonstrated that probiotic supplementation is safe during pregnancy and can significantly reduce nausea, vomiting, and constipation, thereby improving the quality of life of pregnant women (26). Additionally, probiotic use during pregnancy may have a protective role against conditions such as preeclampsia (27), vaginal infections, gestational diabetes (28), and even childhood diseases by influencing the newborn’s microbiome (29). The World Allergy Organization also supports probiotic supplementation for pregnant women at high risk of allergies (30).

Furthermore, many parents lack sufficient knowledge about the interactions between probiotics and medications. Although parents are moderately informed about the basic principles of using probiotics with antibiotics, many are not well-informed about the correct time intervals between taking these supplements and possible interactions with other medications. Numerous studies have confirmed that probiotics can alter drug pharmacokinetics and pharmacodynamics, which can lead to improved or reduced therapeutic efficacy, depending on the type of probiotics and medications used (12, 15, 31–34). Notably, this lack of knowledge is not limited to parents. A recent study conducted among university students of medical sciences in Serbia, who represent future healthcare professionals, revealed similar gaps in understanding regarding probiotic interactions with medications (19). These findings indicate a broader educational challenge that requires improvements in curricula and educational programs, both for the general population and for future medical professionals.

Similarly, several studies conducted in different countries have demonstrated significant variation in parental knowledge of probiotics, particularly regarding their definition, safety, and effects. For instance, a study in Turkey found that only 20.2% of mothers were familiar with the term “probiotics,” while 33.1% recognized specific probiotic products (35). In contrast, a study in China reported that while 95.7% of parents had a basic understanding of probiotics, they lacked the ability to distinguish high-quality probiotic products (36). Meanwhile, a survey in Australia found that 52% of parents were familiar with the term “probiotics” (16). These discrepancies suggest that factors such as educational background, socioeconomic status, and regional health campaigns play a crucial role in shaping parental knowledge and attitudes toward probiotics.

The analysis of parents’ attitudes on this issue shows that the majority of parents have moderately positive attitudes toward probiotics, but also harbor certain doubts and divided opinions on some aspects of their use. Similarly, parents’ attitudes toward probiotic use are generally positive in other studies as well, particularly when they perceive benefits for their children’s health (17, 18). The majority of parents agree that the involvement of healthcare professionals in informing about probiotics is necessary, which could contribute to the proper and safe use of probiotics in children. Parents largely recognize the positive impact of probiotics on the immune system and believe their use is safe from birth. However, a moderate level of agreement with the claim that probiotics can have an effect in the prevention of atopic dermatitis and other skin problems indicates some skepticism or insufficient information about these indications. Supplementation with prebiotics and probiotics appears useful for the reduction in the severity of atopic dermatitis. A systematic review and network meta-analysis involving 21 studies indicated that certain probiotic strains, particularly *Lactobacillus rhamnosus GG* and *Lactobacillus paracasei*, likely reduce the risk of developing atopic dermatitis in children (37). Additionally, about half of the respondents express some doubt about the general effectiveness of probiotics, which may stem from various personal experiences or inconsistent recommendations in available literature and expert advice. Nearly half of the parents consider natural sources of bacteria, such as fermented foods, to be a sufficient alternative to probiotic supplements. However, it is important to emphasize that probiotic supplements contain specific strains in concentrated doses, while natural sources of beneficial bacteria typically contain a mixture of strains in smaller amounts, so their effects are not comparable to the targeted use of probiotic supplements for specific indications (38, 39). A large number of respondents share the opinion that children should not use probiotics for extended periods, which corresponds with other findings of the study, where the majority of parents administer probiotics to their children for less than a month. Some studies suggest that at least three months of continuous use is necessary to improve the immune system (40, 41). There is evidence that long-term use of probiotics in children may contribute to reducing the incidence of allergies in early childhood, lowering the risk of atopy, and establishing a foundation for a healthier immune response during the child’s growth and development (42). Furthermore, although specific recommendations for continued probiotic use after completing antibiotic therapy are not clearly defined, some studies suggest that continuing probiotic supplementation could be beneficial for restoring the gut microbiota and supporting the immune system. Consequently, probiotics are often prescribed for 1–3 weeks after the completion of antibiotic therapy (43). The observed patterns in parents’ attitudes reflect the significant role of healthcare professionals as key sources of information and highlight the need for continuous education to reduce uncertainties and ensure the proper use of probiotics in the pediatric population.

The findings suggest a significant association between parents’ knowledge and attitudes toward probiotics and their likelihood of administering probiotics to their children in the past year. Parents with greater knowledge and more positive attitudes were more inclined to use probiotics, confirming the hypothesis that education on probiotics influences the decision to use them. This result is consistent with the study by Bezek et al., which also found a positive correlation between parental knowledge levels and the inclusion of probiotic supplements in children’s diets (16). However, contrasting findings have been reported in other studies. A study in China revealed that lack of knowledge was one of the primary reasons for not using probiotics in children (18). On the other hand, a previous study conducted in Turkey indicated that many mothers continued to administer probiotics to their children despite having insufficient knowledge about their benefits and appropriate use (35). Similarly, another study conducted in China did not identify a significant correlation between knowledge and practice scores related to probiotics (36). These discrepancies highlight the complexity of factors influencing probiotic use and suggest that, beyond knowledge, cultural practices, healthcare recommendations, and personal beliefs may also play a role in parental decision-making.

The results show a weak negative correlation between probiotic use and the child’s age, suggesting that younger children were more frequently given probiotics. Similar results were obtained in a study conducted in Slovenia, where the highest prevalence of probiotic consumption was also observed among children aged between one and two years (16). This higher use of probiotics in younger children can be partially explained by the more frequent use of antibiotics in this age group (44), as probiotics are recommended as adjunct therapy to preserve the gut microbiota and reduce the side effects of antibiotic treatment. According to the European Society for Paediatric Gastroenterology, Hepatology and Nutrition (ESPGHAN), specific strains such as *Lactobacillus rhamnosus* GG and *Saccharomyces boulardii* are recommended for the prevention of antibiotic-associated diarrhea in children (45). In line with this, the most common reason cited by parents for giving probiotics to their children was concurrent use with antibiotics. The association between probiotic use and health issues in the past year indicates that parents of children who used probiotics during this period most frequently reported gastrointestinal problems, respiratory infections, skin issues, and allergic reactions. It is important to note that, given the cross-sectional design of the study, the observed association between probiotic use and reported health problems likely reflects reverse causation. That is, parents may be more likely to administer probiotics to children who are prone to infections or gastrointestinal issues, rather than probiotics contributing to increased incidence of such problems.

Generally, the most common indication for probiotic use reported in the study is for treating gastrointestinal problems, while their use for strengthening immunity is also common, though to a lesser extent. Furthermore, respondents indicated that they used probiotics less frequently for skin problems, allergic reactions, and respiratory issues, reflecting their lower awareness of the potential benefits for these conditions. Similarly, in a study conducted by Bezek et al., the primary reasons for introducing probiotics were digestive tract disorders, with antibiotic treatment being the most frequent factor (16). In contrast, an Australian study found that the most common reason for probiotic use was the improvement of overall health (54%), with half (51%) of parents believing that probiotics enhanced their child’s general well-being (17). In a study conducted in Denmark, however, parents were generally skeptical about the preventive use of probiotics, often perceiving them as a medicine necessary only when the child is ill (46).

The results also indicate a correlation between the prevalence of medication use and probiotic use in the last year, suggesting that parents of children with more health issues may be more inclined to administer probiotics in an effort to improve their children’s health or to complement medications, particularly alongside antibiotics and for mitigating gastrointestinal side effects (47). In this regard, parents of children who did not use probiotics rated their children’s immunity as significantly higher than those whose children used probiotics. One possible explanation is that parents are more likely to turn to probiotics when they perceive their child to have a weaker immune system, due to the previously discussed role of probiotics in the immune response.

Interestingly, a significant portion of parents stated that they do not pay attention to the composition of the probiotics they give their children, which is consistent with reported lack of awareness about the different effects of various strains. A rapid review of clinical evidence found that while the concept of strain-specificity is widely accepted, there is insufficient evidence to support the claim that probiotic effects in children are strain-specific (48). This lack of evidence may contribute to parents’ confusion and lack of awareness about the importance of specific strains. Parents may choose probiotic products based on general health claims rather than targeting specific health needs, potentially missing opportunities to address particular health issues. For instance, a study conducted in Australia found that parents primarily used probiotics to promote general health, rather than for treating specific conditions (17). This approach may limit the effectiveness of probiotic use in children, as the benefits of probiotics are often strain-specific and may vary depending on the health issue being addressed.

However, most parents rely on recommendations from physicians and pharmacists when selecting probiotics. Nevertheless, nearly a fifth of parents reported that they never consult a doctor before giving probiotics, while the majority do so occasionally or regularly. Similar findings were observed in a study conducted by Bezek et al., where, although many parents followed healthcare professionals’ recommendations, almost 20% administered probiotics without prior consultation (16, 35).

Most parents did not notice any side effects from probiotics in their children; however, a small percentage reported gastrointestinal issues such as constipation, bloating, and diarrhea. These side effects are known to occur even in healthy individuals and children, though they remain relatively rare. While probiotics are widely considered safe for the general population, potential risks, albeit uncommon, should not be overlooked (43, 49). Theoretical concerns include systemic infections, excessive immune stimulation, and horizontal gene transfer (50, 51). These risks become more relevant in specific high-risk groups, such as preterm infants, immunocompromised individuals, elderly persons, and patients with underlying chronic conditions, where cases of bacteremia, endocarditis, or fungemia have been reported (52).

Regarding the concurrent use of probiotics with other medications, most parents administer them separately, indicating some awareness of potential interactions. However, due to the general lack of information and understanding about the potential interactions between probiotics and medications, as previously discussed, additional education on this topic would be highly beneficial.

The binary logistic regression analysis revealed that parents with higher education levels and those from healthcare professions have significantly greater odds of demonstrating good knowledge about probiotics. Additionally, parents who administer probiotics to their children for more than 10 days and those who consider probiotic strain selection are more likely to have sufficient knowledge and positive attitude toward probiotics. In contrast, parental age and the number of children do not significantly influence probiotic knowledge. Similarly, a cross-sectional study conducted in Turkey found that higher education levels were associated with better knowledge of probiotics, along with other factors such as employment, income, and family structure (35). However, unlike our findings, a study in China reported that parents of older preschool children (ages 5–6) were more likely to have positive perceptions of probiotic supplements, suggesting that a child’s age may play a role in shaping parental attitudes in certain populations (18). These variations highlight the influence of sociocultural and demographic factors on parental knowledge and perceptions of probiotics.

The strength of this research lies in being the first known study in Serbia to examine the knowledge, attitudes, and practices of parents regarding probiotic use in preschool children. An additional strength of the study is the large sample size, which allows for more reliable conclusions and analyses. However, several limitations should be acknowledged. First, the cross-sectional design of the study precludes any causal inferences between the examined variables. Second, the data is based on self-reports from parents, which carries the risk of subjective responses, including recall bias and social desirability bias, potentially leading to over- or under-reporting of actual knowledge and practices. Third, although efforts were made to disseminate the questionnaire through multiple social media platforms across different regions of Serbia, there is a possibility that not all regions or population groups were equally represented, which may limit the overall generalizability of the findings. Despite these limitations, the obtained results can serve as a significant foundation for further, more detailed and broader research on this topic, as well as for the creation of educational interventions aimed at improving parental awareness of probiotic use in children.

## 5 Conclusions

The obtained results provide valuable insight into the knowledge, attitudes, and practices of parents regarding probiotic use in children, highlighting both areas of good understanding and those that require further education. The study revealed that the majority of parents of preschool children in Serbia possess fair knowledge and attitudes about probiotics, with significant uncertainties regarding their use, composition, effects, and interactions with other medications, where the lowest level of awareness was observed. This lack of awareness may lead to inappropriate use or missed opportunities for targeted health benefits. The observed correlation between knowledge, attitudes, and probiotic practice emphasizes the importance of education and underscores the need for increased involvement of healthcare professionals in providing accurate information and guidance. The obtained results suggest that healthcare professionals are key trusted sources of information and could serve as effective channels for educational interventions aimed at improving parental knowledge and use of probiotics. Specifically, there is a need for more attention to the preventive use of probiotics and strain-specific effects. This would help ensure the proper and safer application of probiotics in the pediatric population.

## Data Availability

The raw data supporting the conclusions of this article will be made available by the authors, without undue reservation.
