# Supplementary material for: Parental knowledge, attitudes, and practices on probiotic use in preschool children in Serbia: a cross-sectional study
Source: Front Immunol. 2025 Sep 1;16:1601876. doi: 10.3389/fimmu.2025.1601876 (PMC12433886; doi:10.3389/fimmu.2025.1601876)
Supplement: Supplementary file 1 [file DataSheet1.pdf]

Supplementary File. Survey Questionnaire (English)

PART I – Sociodemographic and general information

1. **Parent's gender:**
   - Mother
   - Father

2. **How old are you?**
   - 18-25
   - 26-30
   - 31-35
   - 36-40
   - 41-45
   - Over 45

3. **What is your highest level of education?**
   - Primary school
   - High school
   - Higher vocational education (non-university degree)
   - Bachelor's or master's degree (university degree)
   - Postgraduate education (PhD, specialization, etc.)

4. **What is your place of residence?**
   - Urban area
   - Rural area
   - Suburban area

5. **What is your employment status?**
   - Employed
   - Unemployed
   - Student

6. **Are you a healthcare professional (e.g., physician, pharmacist, dentist, nurse)?**
   - Yes
   - No

7. **What is the number of children in your family?**
   - 1
   - 2
   - 3
   - 4
   - 5 or more

8. **What is the gender of the child for whom you are filling out the questionnaire?**
   - Male
   - Female

9. **What is the age of the child?** (please specify) _____

10. **How would you rate your child's immunity?**
    - Very good
    - Quite good
    - Average
    - Quite weak
    - Very weak

12. **Has your child experienced any of the following health problems in the past 12 months? (check all that apply)**

- Gastrointestinal issues *(vomiting, constipation, diarrhea, bloating, intestinal parasites, bacterial and viral infections of the intestines, etc.)*
- Colds and other respiratory infections *(runny nose, cough, pneumonia, etc.)*
- Suspected or confirmed COVID-19 infection
- Skin problems *(eczema, atopic dermatitis, rash, urticaria, etc.)*
- Allergic respiratory reactions
- Bone fractures, injuries
- No health problems
- Other (please specify)_________

13. **Which of the following medications have you given your child in the past 12 months? * (check all that apply)**

- Antibiotics
- Antiparasitic drugs
- Antihistamines (allergy medications)
- Corticosteroids for local use (inhalers, creams, ointments)
- Corticosteroids for systemic use (injections, tablets)
- Local respiratory medications (inhalers and nasal drops)
- Fever reducers, pain relievers, and anti-inflammatory medications
- Cough syrups
- Other (please specify) ____
- I have not given any medications

* Examples of commonly used licensed drugs in Serbia were included in parentheses to help parents identify each medication group

14. **Which of the following supplements have you given your child in the past 12 months? (check all that apply)**

- Multivitamins
- Vitamin D
- Vitamin C
- Probiotics
- Omega-3 fatty acids
- Minerals
- Immune system supplements
- Other (please specify)____________
- I have not given any supplements

* Examples of commonly used supplements in Serbia were included in parentheses to help parents identify each group

15. **How would you rate your knowledge of probiotics?**

- I am well familiar with probiotics and their effects
- I have basic knowledge of probiotics
- I know a little about probiotics but am not fully aware of their effects
- I know very little about probiotics
- I have almost no knowledge of probiotics

16. **Have you ever given probiotics to your child?**

- Yes
- No

*If you answered **NO**, please go to **PART III***

PART II – Experience with the use of probiotics in children

(*to be filled out only by parents who answered "Yes" to question 16*)

17. In which situations do you usually give probiotics to your child? (check all that apply)

- o During antibiotic therapy
- o Gastrointestinal issues (diarrhea, constipation, nausea, vomiting, intestinal infections, etc.)
- o Respiratory issues (cold, cough, runny nose, pneumonia, etc.)
- o Prevention and strengthening immunity
- o Prevention and treatment of allergic reactions
- o Prevention and reduction of skin disease symptoms (atopic dermatitis, eczema, etc.)
- o Other (please specify) _____

18. What is the longest period you have given probiotics to your child continuously?

- o Less than 10 days
- o 10-30 days
- o 1-3 months
- o 4-6 months
- o More than 6 months

19. Do you consult your physician or pediatrician before giving probiotics to your child?

- o Always
- o Sometimes
- o Never

20. How do you choose probiotics for your child?

- o Based on doctor's recommendation
- o Based on pharmacist's recommendation
- o Based on the composition of the probiotic
- o Based on price
- o Based on brand/manufacturer
- o Other (please specify) ___

21. Which probiotic composition do you most often give/have given to your child?

- o Lactobacillus
- o Bifidobacterium
- o Saccharomyces boulardii
- o Combination of strains
- o I don't pay attention
- o Other (please specify) ____

22. Have you noticed any side effects while giving probiotics to your child?

- o Yes (please specify) ___________
- o No

23. Have you ever given probiotics to your child while taking other medications (other than antibiotics)?

- o Yes
- o No

*If you answered **NO**, please go to **PART III***

24. If you answered yes to the previous question, how did you administer the probiotic?

- o At the same time as the other medication (concurrently)
- o At a different time from the medication (separately)
- o I did not pay attention

PART III – Knowledge on probiotics

For each of the following statements, mark whether you think it is true or false.

	True	False	Unsure
Probiotics are live microorganisms that confer health benefits to the host when administered in adequate amounts	*		
All probiotic strains are expected to have the same or similar effects		*	
Probiotic bacteria in the gut play a crucial role in supporting a child's immune system	*		
The use of probiotics during pregnancy is generally considered safe	*		
Breast milk contains beneficial bacteria	*		
Probiotics may reduce the therapeutic effects of certain medications when taken concurrently	*		
Probiotics may enhance the therapeutic effects of certain medications when taken concurrently	*		
Probiotics are used with antibiotic therapy to enhance their effect		*	
Probiotics can reduce the side effects of antibiotics	*		
The best effect is achieved if antibiotics and probiotics are taken concurrently during the day		*	

** represents the correct answers*

PART IV – Attitudes towards probiotics

For each of the following statements, mark the extent to which you agree.

	Strongly agree	Agree	I'm not sure	Disagree	Strongly disagree
I believe probiotics are safe for use in children from birth					
I am skeptical about the effectiveness of probiotics					
I believe natural sources of beneficial bacteria (e.g., yogurt) can adequately replace probiotic supplements					
I believe probiotics should not be used daily in children for prolonged periods					
I believe probiotics have a positive impact on children's immune systems					
I believe probiotics are effective in preventing atopic dermatitis, eczema, and other skin conditions					
I believe healthcare professionals should play a greater role in educating parents about the use of probiotics in children					
